# A comprehensive analysis of COVID-19 transmission and mortality rates at the county level in the United States considering socio-demographics, health indicators, mobility trends and health care infrastructure attributes

**DOI:** 10.1371/journal.pone.0249133

**Published:** 2021-04-01

**Authors:** Tanmoy Bhowmik, Sudipta Dey Tirtha, Naveen Chandra Iraganaboina, Naveen Eluru

**Affiliations:** Department of Civil, Environmental & Construction Engineering, University of Central Florida, Orlando, Florida, United States of America; La Trobe University, AUSTRALIA

## Abstract

**Background:**

Several research efforts have evaluated the impact of various factors including a) socio-demographics, (b) health indicators, (c) mobility trends, and (d) health care infrastructure attributes on COVID-19 transmission and mortality rate. However, earlier research focused only on a subset of variable groups (predominantly one or two) that can contribute to the COVID-19 transmission/mortality rate. The current study effort is designed to remedy this by analyzing COVID-19 transmission/mortality rates considering a comprehensive set of factors in a unified framework.

**Methods and findings:**

We study two per capita dependent variables: (1) daily COVID-19 transmission rates and (2) total COVID-19 mortality rates. The first variable is modeled using a linear mixed model while the later dimension is analyzed using a linear regression approach. The model results are augmented with a sensitivity analysis to predict the impact of mobility restrictions at a county level. Several county level factors including proportion of African-Americans, income inequality, health indicators associated with Asthma, Cancer, HIV and heart disease, percentage of stay at home individuals, testing infrastructure and Intensive Care Unit capacity impact transmission and/or mortality rates. From the policy analysis, we find that enforcing a stay at home order that can ensure a 50% stay at home rate can result in a potential reduction of about 33% in daily cases.

**Conclusions:**

The model framework developed can be employed by government agencies to evaluate the influence of reduced mobility on transmission rates at a county level while accommodating for various county specific factors. Based on our policy analysis, the study findings support a county level stay at home order for regions currently experiencing a surge in transmission. The model framework can also be employed to identify vulnerable counties that need to be prioritized based on health indicators for current support and/or preferential vaccination plans (when available).

## Introduction

Coronavirus disease 2019 (COVID-19) pandemic, as of August 20^th^, has spread to 188 countries with a reported 23.1 million cases and 802 thousand fatalities [1]. The pandemic has affected the mental and physical health of people across the world significantly taxing the social, health and economic systems [2, 3]. Among the various countries affected, United States has reported the highest number of confirmed cases (5.5 million) and deaths (173 thousand) in the world [4]. In this context, it is important that we clearly understand the factors affecting COVID-19 transmission and mortality rate to prescribe policy actions grounded in empirical evidence to slow the spread of the transmission and/or prepare action plans for potential vaccination programs in the near future. Towards contributing to these objectives, the current study develops a comprehensive framework for examining COVID-19 transmission and mortality rates in the United States using COVID-19 data at a county level encompassing about 93% of the US population. The study effort is designed with the objective of including a universal set of factors affecting COVID-19 in the analysis of transmission and mortality rates. We employ an exhaustive set of county level characteristics including (a) socio-demographics, (b) health indicators, (c) mobility trends, and (d) health care infrastructure attributes. We recognize that analysis of COVID-19 data without including potentially important factors, as has been the case with earlier work, is likely to yield incorrect/biased estimates for the factors considered. The framework proposed for understanding and quantifying the influence of these factors can allow policy makers to (a) evaluate the influence of population behavior factors such as mobility trends on virus transmission (while accounting for other county level factors), (b) identify priority locations for health infrastructure support as the pandemic evolves, and (c) prioritize vulnerable counties across the country for vaccination (when available).

In recent months, a number of research efforts have examined COVID-19 data in several countries to identify the factors influencing COVID-19 transmission and mortality. Given the focus of our current study, we restrict our review to studies that explore COVID-19 transmission and mortality rate at an aggregated spatial scale. To elaborate, these studies explored COVID-19 transmission and mortality rates at the national [5–8], regional [9, 10], state [11], county [6, 12–16], city [17] and zip code levels [18]. A majority of these studies considered transmission rate as the response variable (transmission rate per capita). The main approach employed to identify the factors affecting the response variables is the linear regression approach. In their analysis, researchers employed a host of independent variables from four variable categories: socio-demographics, health indicators, mobility trends and health care infrastructure attributes. For socio- demographics, studies found income, race and age distribution have a positive association with the COVID-19 transmission [13, 18–20]. Regarding health indicators, earlier research found that smokers, obese and individuals with existing health conditions are more likely to be severely affected by COVID-19 [13]. In terms of mobility trends, studies showed that staying at home and effective mobility restriction measures significantly lower the COVID-19 transmission rate [6, 9, 12, 16, 21–23] while increased mobility resulted in increased COVID-19 transmission [14, 24]. Finally, among health care infrastructure attributes, testing rate is linked with reduced risk of COVID-19 transmission [21, 25]. While earlier research efforts have considered the factors from all variable categories, it is important to recognize that each individual study focused only on a subset of variable groups (predominantly one or two) and have not controlled explicitly for other variable groups that can contribute to the COVID-19 transmission/mortality rate.

The current study builds on earlier literature examining the factors affecting COVID-19 transmission and mortality rate and contributes along the following directions. *First*, we extensively enhance the spatial and temporal coverage of COVID-19 data in our analysis. *Spatially*, earlier research on COVID-19 aggregate data analysis has focused on a small number of counties (up to 100 counties). In our study, we consider all counties with total number of cases greater than 100 on August 4^th^. The 1,752 counties selected encompass 93% of the total population and 95% of the total confirmed COVID-19 cases. *Temporally*, earlier research has only considered data up to the month of April. While these studies are informative, cases in the US grew substantially in the recent months. Hence, in our study we have considered data from March 25^th^ to August 4^th^, 2020. The longer period of data (133 days) also enables us to study/test for the evolution of variable effects over time. *Second*, earlier research studies have considered factors from one or two of the categories of variables identified above. Further, studies that tested health indicators employed one or two measures selectively. In our analysis, we conduct a comprehensive examination of factors affecting COVID-19 from all four categories of variables including (a) socio-demographics: distribution by age, gender, race, income, location (urban or rural), education status, income inequality and employment, (b) health indicators: percentage of population suffering from cancer, cardiovascular disease, hepatitis, Chronic Obstructive Pulmonary Disease (COPD); diabetes, obesity, Human Immunodeficiency Virus (HIV), heart disease, kidney disease, asthma; drinking and smoking habits, (c) mobility trends: daily average exposure, social distancing matrices, percentage of people staying at home, and (d) health care infrastructure attributes: hospitals per capita, ICU beds per capita, COVID-19 testing measures. *Finally*, the research study employs a robust modeling framework in terms of model structure and dependent variable representation. A mixed linear model system that addresses the limitations of the traditional linear regression framework for handling repeated measures is employed. For dependent variable, alternative functional forms of COVID-19 transmission—natural logarithm of daily cases per 100 thousand people and natural logarithm of 7-day moving average of cases per 100 thousand people—are considered in model estimation. The overall approach allows us to robustly quantify the impact of factors affecting COVID-19 transmission.

## Methods

### Data collection

#### Independent variables

**Fig 1:**
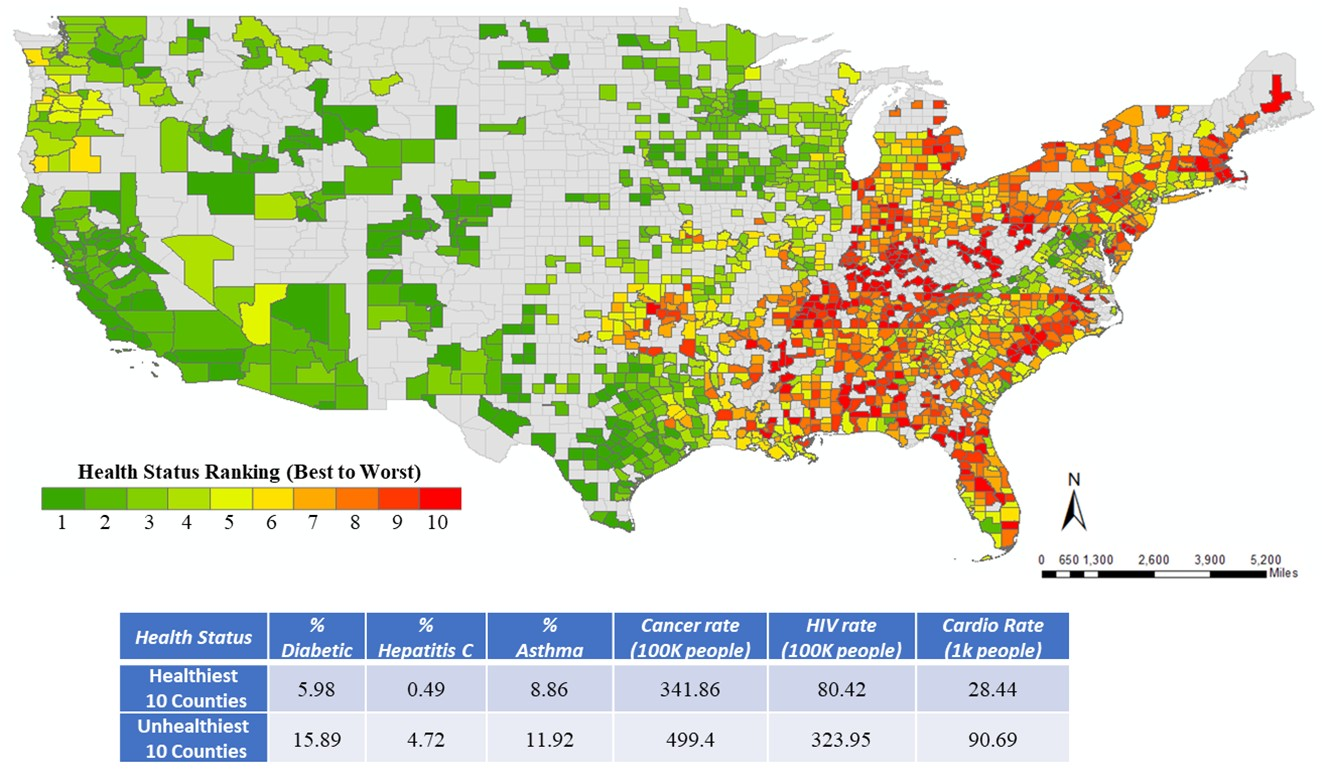
Ranking of counties based on health indicators. *Source US County Map* [28].

Table 1 summarizes sample characteristics of the explanatory variables with the definition considered for final model estimation, the data source, and sample characteristics (minimum, maximum and mean values). The socio-demographic variables are collected from the American Community Survey (ACS) while information on the health indicator variables are gathered from the Centers for Disease Control and Prevention (CDC) systems. Using health indicator data, we ranked the 1,752 counties in a descending order of health metric and provided it in Fig 1. We performed ranking of the counties using multi-criteria decision analysis approach [26–28]. Details on this approach are summarized in the S1 File. Further, we compute the average values for different health indicators across the healthiest and unhealthiest 10 counties (source: US County Map, [29]) to highlight the change in health conditions across the two groups. The values clearly emphasize the vulnerability of the unhealthiest counties relative to the healthiest counties. For instance, number of Cardio patients in the healthy counties are 28.44 while in the unhealthiest counties, it is almost 219% higher (90.69).

**Table 1:** Descriptive statistics of the dependent and independent variables.

Variables	Source	Mean	Min/Max	Sample Size
**Independent Variables**				
***Demographic Characteristics***				
Percentage of population aged 18 years and lower	ACS^a^	22.558	7.155/35.987	1752
Percentage of population aged 65 years and over	ACS	17.256	6.724/56.944	1752
Percentage of African American	ACS	10.994	0.113/80.507	1752
Percentage of Hispanic	ACS	10.344	0.623/96.323	1752
Percentage of Female	ACS	50.386	37.041/54.495	1752
Ln (Median income)	ACS	10.872	10.149/11.822	1752
Percentage of people less than high school education	ACS	14.143	3.127/47.053	1752
Employment rate per capita	ACS	0.441	0.190/0.640	1752
Income inequality ratio (80^th^ percentile/20^th^ percentile)	CHRR^b^	4.547	2.988/9.148	1752
***Health Indicators***				
Ln (HIV Prevalence Rate per 100K people)	CHRR	4.870	0.723/7.859	1752
Hepatitis B Cases per 100K people in2017	CDC^c^	1.338	0.000/11.700	1752
Hepatitis C Cases per 100K people in2017	CDC	1.016	0.000/5.600	1752
Asthma % for > = 18 years	CDC	9.332	7.400/12.300	1752
COPD % for > = 18 years	CDC	6.757	3.300/13.700	1752
Reported cancer case per 100K people	CDC	455.651	241.000/623.000	1752
Percentage of diabetic	CHRR	11.527	3.300/20.400	1752
Percentage of obesity among adults	CHRR	31.951	13.600/46.700	1752
Cardiovascular Disease Hospitalization Rate per 1,000 Medicare Beneficiaries	CDC	63.462	0.300/115.800	1752
***Mobility Trends***				
Ln (Daily Average Exposure), 10 days lag				
From April 25th	CEI^d^	4.176	0.591/7.048	233,016
% People staying at home				
14 days lag	Safegraph	0.143	0.037/0.364	233,016
***Healthcare Related Attributes***				
Hospitals per 100K people	CHRR	2.372	0.000/15.640	1752
Number of ICU beds per capita	CHRR	18.334	0.000/171.850	1752
Ln (No of tests with 5 days lag)	CTP^e^	8.431	0.000/12.015	6,783
***Temporal Factors***				
Day is weekend	--	0.285	0.000/1.000	233,016
**Dependent Variables**				
Ln (Daily COVID-19 transmission rate per 100K people)	CSSE^f^	1.470	0.000/7.668	233,016
Ln (Total COVID-19 mortality rate per 100K people)	CSSE	2.849	0.000/7.237	1752

^a^ = American Community Survey

^b^ = County Health Rankings & Roadmaps

^c^ = Central for Disease Control System

^d^ = COVID Exposure Indices [25]

^e^ = COVID-19 Tracking Project [26]

^f^ = Center for Systems Science and Engineering Coronavirus Resource Center at Johns Hopkins University [27]

To incorporate mobility trends, we considered two variables: daily average exposure and social distancing metric to serve as a surrogate measure for the mobility patterns. The exposure variables provide information compiled based on smartphone movement data within and across the counties in US [30]. For our analysis, we confined our attention to the overlapping movements within the counties. From the movement data provided by PlaceIQ, for each smartphone device visiting a location, the total number of distinct devices visiting that location at that particular time is calculated [30]. These distinct devices will serve as exposure for the particular device. Similarly, one can compute the exposure for all the devices residing in a county and finally compute the daily average exposure at the count level. The reader would note that smartphone movement data is reported for counties with at least 1000 active devices in a day. The 1752 counties selected for analysis satisfied the requirement of minimum active devices.

**Fig 2:**
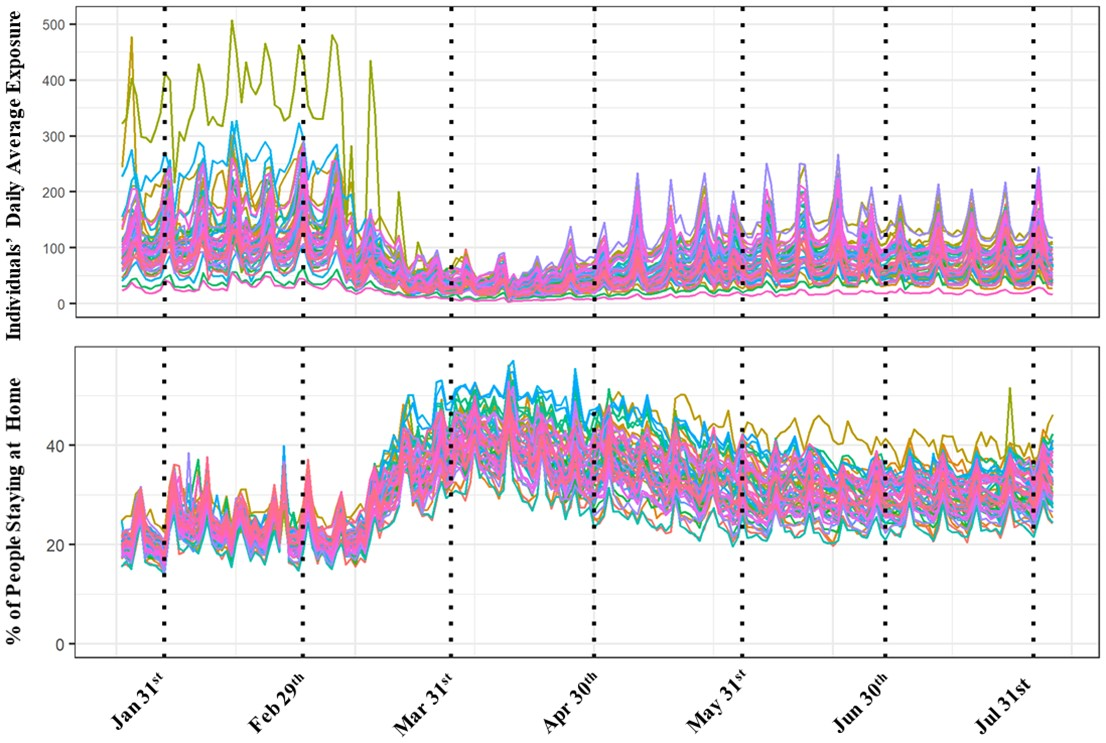
Average daily exposure and percentage of people staying at home.

The second measure, information on social distancing is collected from Safegraph data (see Acknowledgement section for description of Safegraph data). These metrics provide information on the number of devices completely staying at home, mean/median distance travel from home, full time and part time work behavior at a daily basis for each county. Fig 2 provides a summary of both these measures at a state level from January 22^nd^ to August 4^th^. From the figure, we can clearly see the reduction in average daily exposure in March as many states and local jurisdictions imposed lockdowns. By late April, exposure activity started to increase again across all the states while still being lower than the levels for February. In terms of the staying at home measure, as expected, we find an exactly opposite trend.

Finally, within the healthcare infrastructure attributes, information about the hospitals and ICU beds are gathered from the County level health ranking data. COVID-19 testing measures are sourced from the COVID-19 tracking project [31] that provides a complete picture of testing as well the number of positive and negative cases for each county in the United States.

#### Dependent variables

We analyze two county level dependent variables: (1) COVID-19 daily transmission rate per 100K population and (2) COVID-19 mortality rates per 100K population. For the transmission rate analysis, we tested two alternative functional forms: daily cases per 100 thousand people and 7-day moving average of cases per 100 thousand people. The moving average data is likely to be less volatile and serves as a stability test for the daily cases model. The reader would note that we used a natural logarithmic transformation for all the dependent variables. The COVID-19 dataset from Center for Systems Science and Engineering (CSSE) Coronavirus Resource Center at Johns Hopkins University [32] provides information on the daily confirmed COVID-19 cases, number of people recovered (when available) and the number of deaths from COVID-19 starting from January 22^nd^ to the current date across 3,142 counties in the United States. In our research, we confined our analysis to the cases between March 25^th^ to August 4^th^ resulting in 133 days of data. Further, we focus on counties that have at least 100 cases by August 4^th^ and have available information on the mobility trends. With this requirement, a total of 1,752 counties are included in the analysis providing a coverage of 93% of the total population in the United States. For mortality rate, we considered the fatalities within the same time frame across all the 1,752 counties as the transmission rate variable. The summary statistics of the dependent variable are presented in bottom row panel of Table 1.

### Data analysis (Modeling framework)

The two dependent variables: (a) COVID-19 daily transmission rate and (b) COVID-19 mortality rate are continuous in nature and linear regression model is the most traditional method to study such continuous responses. For the analysis of daily transmission rate, we have repeated measures of the variable (133 repetitions for each county). The traditional linear regression model is not appropriate to study data with multiple repeated observations [33]. Hence, we employ a linear mixed modeling approach that builds on the linear regression model while incorporating the influence of repeated observations from the same county. By adopting the linear mixed model, we recognize the dependencies across COVID-19 cases occurring for the same county. A brief description of the linear mixed model is provided below:

Let *q = 1*, *2*, *…*, *Q* be an index to represent each county, and *d = 1*, *2*, *…*, *D* be an index to represent the various days on which data (cases) was collected. The general form of the mixed linear regression model has the following structure:

$$y_{qd}=\beta X+\varepsilon_{qd}$$ (1)

where *y*_*qd*_ is the dependent variable representing the new COVID 19 cases per 100K population, *X* is the vector of attributes and *β* is the model coefficients. *ε*_*qd*_ is the random error termassumed to be normally distributed across the dataset.

This *ε* term captures the dependencies across the repetition for each county. In our analysis, we estimate the correlation for different level of repetition measures: correlation for all records (133 repetitions), monthly level (31 repetitions) and weekly level (7 repetitions). The flexibility offered by the mixed model for testing dependencies enhances the model development exercise over its simpler form. In this structure, the data can be visualized as K (K = 133 or 31 or 7) records for each 1,752 counties. Estimating a full covariance matrix (up to 133*133) is computationally intensive while providing very little intuition. Hence, we parameterize the covariance matrix (Ω).

For estimating a parsimonious specification, we tested first-order autoregressive (AR) and autoregressive moving average (ARMA) correlation structure within the mixed linear model. The reader would note that the final model was identified based on three criteria: autocorrelation function (ACF); a partial autocorrelation function (PACF) and Bayesian Information Criterion metric (BIC). All of these measures provide support to the ARMA model selection (see S1 File for more details). Therefore, in the current study, we will only discuss the framework for the ARMA model (due to space constraints). The ARMA correlation structure comprises three parameters σ, ρ, and φ as follows:

$$\Omega =\sigma^{2}(\begin{array}{ccccc} 1 & \varphi \rho & \varphi \rho^{2} & \cdots & \varphi \rho^{K-1}\\ \varphi \rho & 1 & \cdots & \cdots & \cdots \\ \vdots & \vdots & \vdots & \vdots & \vdots \\ \varphi \rho^{K-1} & \cdots & \cdots & \cdots & 1 \end{array})$$ (2)

where, σ represents the error variance of ε, φ represents the common correlation factor across time periods K, ρ represents the dampening parameter that reduces the correlation with time and K represents the level of repetition. The correlation parameters φ and ρ, if significant, highlight the impact of county effects on the dependent variables. The models are estimated in SPSS using the restricted maximum likelihood estimation (RMLE) approach. For modeling the COVID 19 mortality rate, we rely on simple linear regression approach as the dependent variable here is the total number of COVID-19 deaths per 100K population at a county level.

## Results

The reader would note that prior to estimating the models, we checked for the multicollinearity issue across the independent variables as it is possible that county level characteristics are highly correlated. We did not find any significant impact of multicollinearity on our model estimates (see S1 File for more details).

### COVID-19 transmission rate model results

The estimation results for the linear mixed model are presented in Table 2. From this point, we will use the term transmission rate for representing the natural logarithm of daily COVID-19 cases per 100K population. As discussed earlier, we also developed the same mixed linear model to estimate the 7-day moving average of COVID-19 cases per capita and find similar results as in the daily COVID-19 transmission model (results are available upon request from the authors). This further reinforces the stability of the transmission model.

**Table 2:** Estimation results for daily COVID-19 transmission rate per 100K population.

Variables	*Estimates*	*t-statistic*	*p-value*
Constant	-4.882	-18.307	<0.001
***Demographics***			
% of Female population	0.019	8.794	<0.001
% Young population (< = 18 years)	0.009	6.097	<0.001
% of African-American population	0.010	27.055	<0.001
% of People less than high school education	0.022	22.738	<0.001
Ln (median income)	0.325	14.185	<0.001
Employment rate per capita	0.963	9.320	<0.001
Ln (% of People living in rural areas)	-0.408	-17.567	<0.001
***Health Indicators***			
Ln (HIV rate per 100K People)	0.044	7.441	<0.001
Hepatitis C rate per 100K People	0.012	3.200	<0.001
***Mobility Trends***			
Ln (Daily Average Exposure), 10 days lag			
April 25^th^ to July 21^st^	0.028	12.360	<0.001
After July 21^st^	0.171	17.085	<0.001
% People staying at home (14 days lag)			
March 25^th^ to July 21^st^	-0.590	-5.564	<0.001
After July 21^st^	-3.952	-13.023	<0.001
***Health Care Infrastructure Attributes***			
Ln (Testing), 5 days lag			
March 25^th^ to May 10th	0.012	7.654	<0.001
After May 10th	0.019	15.350	<0.001
***Temporal Factors***			
Temporal Lagged Variables			
7 days lag (March 25^th^ to June 22^nd^)	0.177	69.165	<0.001
7 days lag (June 23^rd^ to July 6^th^)	0.285	66.121	<0.001
7 days lag (After July 6^th^)	0.362	115.590	<0.001
14 days lag	0.167	77.272	<0.001
Day is Weekend	-0.045	-10.695	<0.001
***Correlation***			
*σ*	0.988	275.252	<0.001
*ρ*	0.959	367.088	<0.001
*ϕ*	0.286	102.854	<0.001

#### Socio-demographics

We find several socio-demographic variables to have significant impact on the transmission rate. In terms of female population, we find that higher proportion of females in the population has a positive impact on transmission rated. At first glance, the result might appear to be contradicting earlier studies that show women are less likely to be affected by COVID-19 transmission relative to men [18]. However, the reader would note that this result only implies that counties with higher percentage of female population are likely to experience increased number of COVID-19 cases relative to other counties. The finding does not necessarily indicate that women are at a higher risk of being infected by COVID-19. For differences in proclivity for COVID-19 infection by gender, individual level data would be a more appropriate avenue for analysis. Among age distribution proportions, we found that increased percentage of younger individuals (<18 years) is associated with more transmission. In terms of racial distributions, counties with higher proportion of African-Americans are likely to have higher transmission rates (see earlier work for similar findings [13, 20]). It has been postulated that African-Americans in general reside in densely populated low income neighborhoods with lower access to amenities and are employed in industries that requires more public exposure [19]. Educational status in a county also plays an important role in influencing the COVID-19 transmission. The counties with higher share of individuals with less than high school education are likely to report increased incidence of COVID-19. In terms of income, we find that higher median income in a county leads to rise in daily COVID-19 incidence. The effect of income might appear counter-intuitive at first glance. However, it is possible that higher income individuals are more likely to get tested (even in the absence of symptoms) due to higher health insurance affordability. Low income individuals are more likely to lose their jobs and health insurance coverage due to COVID-19 pandemic [13, 34]. With respect to employment rate, counties with higher employment rate reflect more exposure and have a positive association with transmission. The percentage of people living in rural area offers a negative association with the daily COVID-19 incidence. This indicates that people living in rural areas are less affected by COVID-19. This is intuitive as rural areas are sparsely populated and hence have more opportunity for social distancing thus lowering transmission rates.

#### Health indicators

With respect to health indicators, we tried several variables in the transmission rate model. Of these, two variables number of people suffering from HIV and hepatitis C in a county offered significant impacts. We observe that counties with higher percentage of HIV and hepatitis C patients have an increased incidence of COVID-19 transmission. Individuals with these diseases have weaker immune systems and hence are more susceptible to COVID-19 transmission.

#### Mobility trends

In terms of mobility trends, we tested two measures: daily average exposure and percentage of people staying at home. In considering these variables in the model, we recognize that exposure will have a lagged effect on transmission i.e. exposure to virus today is likely to manifest as a case in the next 5 to 14 days. In our analysis, we tested several lag combinations and selected the 10 day lag exposure as it offered the best fit. Similarly, for people staying at home, the 14 day lag offered the best fit. The exposure variable offers interesting results. Until April 25^th^ exposure variable does not have any impact on transmission. This is strongly coinciding with the lower exposure trends (see Fig 2). After April 25^th^, increased exposure is associated with higher transmission rates 10 days into the future (see Hamada and colleagues [24] for similar findings). Further, the influence of exposure is substantially larger after July 21^st^ indicating a higher risk of exposure for COVID-19 transmission. For the second measure, staying at home with 14 days lag, we find that daily transmission rates are negatively affected as expected [12, 21]. The impact of staying at home percentage is particularly stronger in recent weeks as indicated by the higher negative impact from July 21^st^. The two variable effects since July 21^st^ reflect the influence of increased exposure to COVID-19 in recent weeks across the country. The reader would note that the two measures considered were not found to be strongly correlated (see S1 File for details) and thus were simultaneously considered in the model.

#### Health care infrastructure attributes

The only set of variables found to have a significant impact of COVID-19 transmission rate within this category correspond to COVID-19 testing effects. Again, we select a 5 day lag as we believe testing results are provided in 3–5 days. The coefficient of this variable is positive as expected and highly significant [21]. However, after May10^th^, the effect has a higher magnitude which suggests that compared to the previous time period (before May 10^th^), higher testing rate will increase the daily COVID-19 transmission at a marginally higher rate.

#### Temporal factors

With data available for 133 days, we can evaluate the effect of the transmission rate in previous time period on the current time period. As expected, we find a positive association between the daily COVID-19 transmission rate and the temporal lagged variables in the previous time period for 7 and 14 days. The result suggests higher transmission rate in previous time periods (7 and 14 days earlier) is likely to result in increased transmission. However, the effect is higher for the 7 day lagged variable, as evidenced by the higher magnitude associated with the corresponding time period in Table 2. Further, the 7 day lagged transmission rate after June 21^st^ and July 7^th^ time period offer larger positive impacts. Unsurprisingly, the effect for July 7^th^ and later is significantly larger than the other variable effect. The result is aligned with the sudden surge in COVID-19 cases since beginning of July. Finally, the weekend variable highlights that the COVID-19 transmission rate is lower during weekends possibly because of reduced testing rate on weekends [35].

#### Correlation

As indicated earlier, we developed the mixed linear model for estimating the daily COVID-19 transmission rate per 100,000 people while incorporating the dependencies across each county for multiple repetition levels. Of these different models, we selected the model that provides best result in terms of statistical data fit and variable interpretation. We found that the model accommodating weekly correlations provided the best result. The final set of variables in Table 2 corresponds to the correlation parameter across every 7 days within a county. All the parameters are highly significant highlighting the role of common unobserved factors influencing the daily COVID-19 transmission rate over a week across the counties.

### COVID-19 mortality rate

As opposed to the transmission rate model, we adopted a simple linear regression approach to study the determinants of the COVID-19 mortality rate at a county level. The coefficients in Table 3 represent the effect of different independent variables on the COVID-19 mortality rate (total number of deaths per 100K population in 3 months period) at a county level.

**Table 3:** Estimation results for COVID-19 mortality rate per 100K population.

Variables	*Estimates*	*t-statistic*	*p-value*
Constant	-6.467	-3.741	<0.001
***Demographics***			
Older people % (>65 years old)	0.053	6.663	<0.001
% of African-American population	0.021	8.077	<0.001
% of People less than high school education	0.070	10.730	<0.001
Income inequality ratio	0.168	3.700	<0.001
Employment rate per capita	6.381	7.953	<0.001
Ln (% of People living in rural areas)	-1.335	-7.061	<0.001
***Health Indicators***			
Ln (HIV rate per 100K people)	0.200	4.889	<0.001
Cancer rate per 100K people	0.256	1.919	0∙036
Hepatitis A rate per 100K People	0.051	2.157	0∙031
Ln (Cardiovascular disease per 1K people)	0.386	3.064	0∙002
***Health Care Infrastructure Attributes***			
ICU beds per capita	-0.007	-4.382	<0.001

#### Socio-demographics

With respect to socio-demographic variables, we find several attributes to have a significant impact on the COVID-19 mortality rate. For instance, higher percentage of older people in a county leads to an increased COVID-19 mortality rate as indicated by the positive coefficient in the Table 3. Similar results are also observed in earlier studies [16, 20]. Further, consistent with previous research [19], the current analysis also found a positive coefficient associated with the percentage of African-American people revealing a higher COVID-19 mortality rate in counties with higher proportion of African-American people. The variable specific to education status indicates that the likelihood of COVID-19 mortality increases with increasing share of people with less than high school education in a county. From the estimated results presented in Table 3, we find that counties with higher income inequality ratio are more likely to experience higher number of COVID-19 deaths per capita relative to the counties with lower income disparities. Higher income inequality mainly reflects a significant share of low-income workers who possibly need to continue their daily activities despite the risk of COVID-19 transmission. Further, they usually have less access to the health care system and thus have an increased risk of mortality [36]. Moreover, we find a positive association between the employment rate and COVID-19 mortality rate in a county. As discussed in the transmission model, high employment rate mainly reflects increased exposure which eventually increases the risk of COVID transmission resulting in higher risk of COVID-19 mortality. Finally, the last variable in the demographic category corresponds to the percentage of people living in rural areas that implies a negative effect on COVID-19 mortality rate indicating a reduced COVID-19 mortality rate in a county with more people living in the rural regions.

#### Health indicators

Among the health indicators, we found several variables significantly influence the COVID-19 mortality rate in a county. For instance, in comparison to other counties, counties with higher number of HIV, cancer, hepatitis A and cardiovascular patients are more likely to have higher number of COVID-19 deaths. This is expected as people with such conditions usually have weaker immune system which makes them vulnerable to the disease. The results are in line with a number of earlier studies [5, 37, 38].

#### Health care infrastructure attributes

Finally, among health care infrastructure attributes, number of ICU beds per capita at a county is found to have a negative impact on COVID-19 mortality rate suggesting a reduced death rate with higher number of ICU bed per person in a county. The result is intuitive as more ICU bed per capita indicates the county is well equipped to handle higher patient demand and treatment is accessible to more COVID-19 patients.

### Policy implications

To illustrate the applicability of the proposed COVD-19 transmission model, we conduct a scenario analysis exercise by imposing hypothetical mobility restrictions. While earlier researchers explored the influence of mobility measures, these models did not account for county level factors such as socio-demographics, health indicators and hospital infrastructure attributes. In our framework, the sensitivity analysis is conducted while controlling for several other factors. The hypothetical restrictions on mobility are considered through the following changes to two variables:

1. county level average daily exposure reduced by 10%, 25% and 50%
2. county level percentage of stay at home population increased to 40%, 50% and 60%.

The changes to the independent variables were used to predict the transformed dependent variable. Subsequently, the transformed variable was converted to the daily cases per 100 thousand people. The results from this exercise are presented in Table 4. We present the average change in cases for all counties (1,752), and for the 100 counties with the highest overall transmission rates. From Table 4, two important observations can be made. First, changes to average daily exposure and stay at home population influence COVID-19 transmission significantly. In fact, by increasing stay at home population share to 50%, the model predicts a reduction of the number of cases by about 33%. Further, mobility restriction results in suppressed COVID-19 transmission as indicated by the negative values from Table 4. Second, the benefit from mobility restrictions and staying at home is slightly higher for the worst 100 counties with higher overall cases. The two observations provide evidence that issuing lockdown orders in counties with a recent surge is a potential mitigation measure to curb future transmission.

**Table 4:** Policy scenario analysis of social distancing in COVID-19 transmission rate per 100K population.

Hypothetical Scenarios	*1*,*752 Counties*	*Worst 100 Counties*
1: daily average exposure reduced by 10%	-0.636	-0.640
2: daily average exposure reduced by 25%	-1.716	-1.726
3: daily average exposure reduced by 50%	-4.030	-4.055
4: 40% people stay at home	-26.423	-26.654
5: 50% people stay at home	-33.082	-33.258
6: 60% people stay at home	-38.561	-38.700

The COVID-19 total mortality rate model can be employed to identify vulnerable counties that need to be prioritized for vaccination programs (when available). While prioritizing the counties based on mortality rate might be a potential approach, it might be feasible. To elaborate, vaccination programs have to be planned well in advance (say 2 months) of the vaccine availability. As total mortality rates for 2 months into the future are unavailable, we need a model to predict total mortality into the future. The estimated mortality rate model provides a framework for such analysis. To be sure, it would be prudent to update the proposed model with the latest data to develop a more accurate prediction system.

## Discussion

The current study develops a comprehensive framework for examining COVID-19 transmission and mortality rates in the United States at a county level including an exhaustive set of independent variables: socio-demographics, health indicators, mobility trends and health care infrastructure attributes. In our analysis, we consider all counties with total number of cases greater than 100 on August 4^th^ and analyze daily cases data from March 25^th^ to August 4^th^, 2020. The COVID-19 transmission rate is modeled at a daily basis using a linear mixed method while the total mortality rate is analyzed adopting a linear regression approach.

Several county level factors including proportion of African-Americans, income inequality, health indicators associated with Asthma, Cancer, HIV and heart disease, percentage of stay at home individuals, testing infrastructure and Intensive Care Unit capacity impact transmission and/or mortality rates. The results clearly support our hypothesis of considering a universal set of factors in analyzing the COVID-19 data. Further we conducted policy scenario analysis to evaluate the influence of social distancing on the COVID-19 transmission rate. The results highlight the effectiveness of social distancing in mitigating the virus transmission. In fact, we found that by increasing stay at home population share to 50% the model predicts a reduction of the number of cases by about 33%. The finding provides evidence that issuing lockdown orders in counties with a recent surge is a potential mitigation measure to curb future transmission.

To be sure, the study is not without limitations. The study is focused on county level analysis and is intended to reflect associations as opposed to causation. However, for the causation based analysis, data from individuals would be more suitable. As with any area level analysis, there is a small possibility that some of the estimated parameters might be spurious associations due to aggregation bias. However, in the absence of individual level data, these area level models offer a valid and useful tool for epidemiologists and planners. Further, the inherent aggregation of the data at a county level would initiate some form of spatial heterogeneity which we did not account for in our analysis. In future, it would be interesting to accommodate these effects separately while considering the temporal correlation. Further, the proposed model can be enhanced using more detailed information such as percentage of health workers in the workforce, number of hospital beds and mask mandate dates. While exposure data were reasonably addressed, data was not available for mask wearing behavior across all counties. Finally, the data on transmission and mortality are updated for few counties to correct for errors or omissions. These were carefully considered in our data preparation. However, it is possible that further updates might be made after we finished our analysis.

## Supporting information

S1 File (PDF)
