# Supplementary material for: A comprehensive analysis of COVID-19 transmission and mortality rates at the county level in the United States considering socio-demographics, health indicators, mobility trends and health care infrastructure attributes
Source: PLoS One. 2021 Apr 1;16(4):e0249133. doi: 10.1371/journal.pone.0249133 (PMC8016225; doi:10.1371/journal.pone.0249133)
Supplement: S1 File — (PDF) [file pone.0249133.s001.pdf]

Multi-criteria decision analysis

We performed ranking of the counties using a multi-criteria decision analysis approach. In this approach, alternatives (or counties) are first scored based on weights and values of the criteria (or health indicators). In our study, we considered the equal weights for six health indicators. Then, health indicator measures are normalized, and each county is scored based on normalized health indicator values using the weighted sum method (1–3). The equation for scoring the counties is provided below:

$$S_j = \sum_{i=1}^{6} HI_{ij} \times W_i \quad (1)$$

Here, S_j is the overall score of county j. HI_{ij} is the normalized value of indicator, i for county j and W_i (=1/6) is the weight of the indicator, i. Finally, the counties are ranked based on county S_j scores. The county with the lowest overall score is ranked as the best county and the county with the highest score is ranked as the worst county.

Model Selection

We examined autocorrelation function (ACF) and a partial autocorrelation function (PACF) for our data. ACF and PACF provide guidance to identify the most appropriate model form between AR, MA and ARMA model alternatives (4). A guide to model selection is presented in the Table below:

Table 5 A guide to Model Selection based on ACF and PACF

Model	ACF	PACF
AR	Geometric	Significant till p lags
MA	Significant till q lags	Geometric
ARMA	Geometric	Geometric

We generated ACF and PACF for our response variable (see Fig 3). From the figure, it is apparent that ACF and PACF function are geometric in nature providing further support to our ARMA model selection.

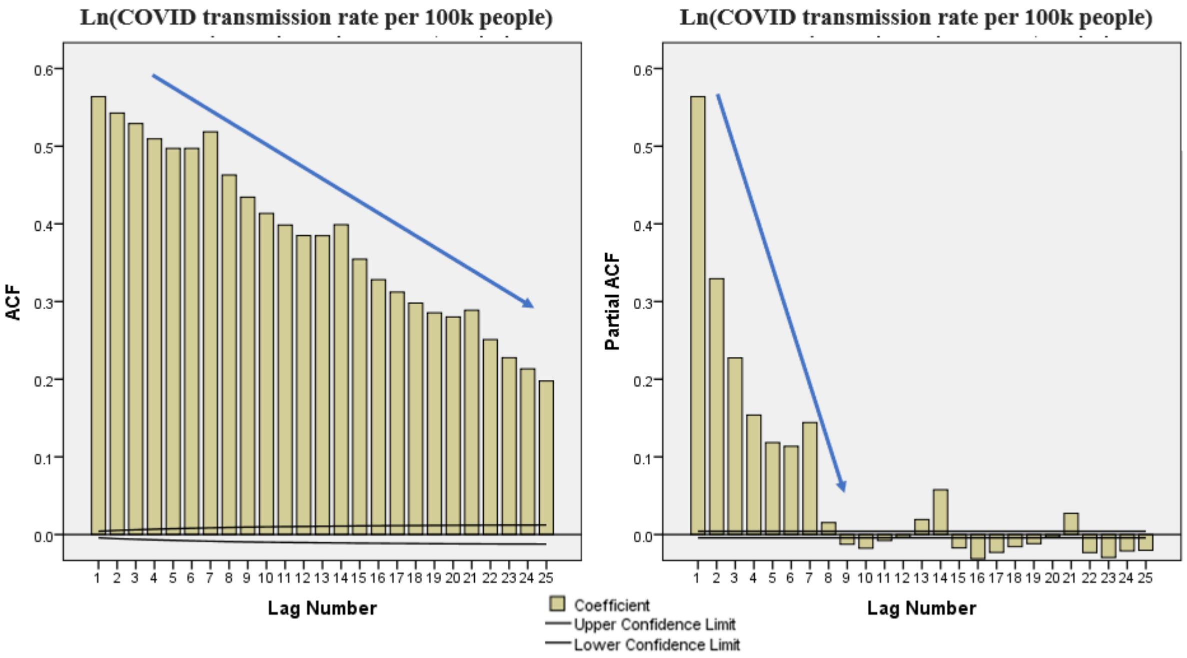

Fig 3: ACF and PACF of the Response Variable

Further, as we have some significant lags in the PACF, we estimated both AR and ARMA model and compared their BIC values. In our case, the BIC values are: AR: 641273.08 and ARMA: 628694.67. The comparison further reinforces our preference of ARMA structure over the AR model.

Correlation and Multicollinearity

We plot a correlation matrix to highlight the Pearson correlation coefficient between each independent variable in the dataset. Fig 4 presents the correlation matrix across the different independent variables found significant in the analysis. The figure clearly highlights the weaker

correlation across majority of the predictors while finding the presence of mild correlations (>0.5 and <0.68) across the following pairs (income, employment rate); (% of People less than high school education and employment rate); (HIV rate and % of African-American); and (% young and senior people).

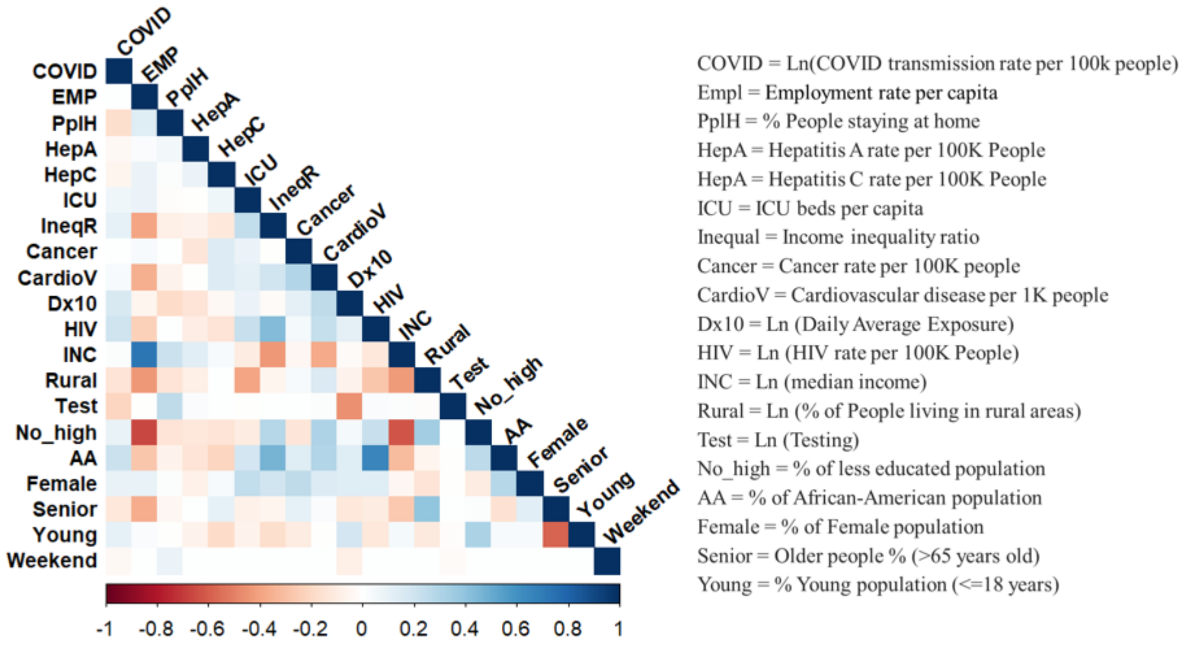

Fig 4 Pearson Correlation Matrix Across the Variables

As the multicollinearity is suspected across the above-mentioned pairs, we estimate the variance inflation factor (VIF) for these variables. VIF estimates the increase in variance of the coefficient for each variable in response to the correlated predictors. A VIF value of 1 for a variable implies complete independence from any other predictors in the model. VIF value greater than 5 is usually considered as a high level of collinearity while less than 5 is believed a non-significant multicollinearity that does not require any correction (5). In our analysis, the maximum value of VIF we found is 3.8 (removing one variable at a time and then replace it: rotating the set of independent variables to arrive at an accurate representation of VIF) and therefore we can safely conclude that our analysis was unaffected by the multicollinearity issue."

REFERENCES

1. Triantaphyllou E, Sánchez A. A sensitivity analysis approach for some deterministic multi-criteria decision-making methods. Decis Sci. 1997;
2. Rehman S, A. S. Multi-Criteria Wind Turbine Selection using Weighted Sum Approach. Int J Adv Comput Sci Appl. 2017;
3. Mateo JRSC. Weighted sum method and weighted product method. In: Green Energy and Technology. 2012.
4. Robinson, G. M.. Time Series Analysis. In International Encyclopedia of Human Geography. 2009; 285–293. Elsevier Inc. https://doi.org/10.1016/B978-008044910-4.00546-0
5. Ringle CM, Wende S, Becker JM. SmartPLS 3. Boenningstedt: SmartPLS GmbH. 2015.
